# Antisense oligonucleotides as a potential treatment for brain deficits observed in myotonic dystrophy type 1

**DOI:** 10.1038/s41434-022-00316-7

**Published:** 2022-01-25

**Authors:** Siham Ait Benichou, Dominic Jauvin, Thiéry De Serres-Bérard, Marion Pierre, Karen K. Ling, C. Frank Bennett, Frank Rigo, Genevieve Gourdon, Mohamed Chahine, Jack Puymirat

**Affiliations:** 1 LOEX, CHU de Québec-Université Laval Research Center, Quebec City, QC Canada; 2 CERVO Research Center, Institut universitaire en santé mentale de Québec, Quebec City, QC Canada; 3 Ionis Pharmaceuticals Inc., Carlsbad, CA USA; 4 Sorbonne Université, Inserm, Association Institut de Myologie, Centre de recherche en Myologie, Paris, France; 5 Department of Medicine, Faculty of Medicine, Université Laval, Quebec City, QC Canada

**Keywords:** Neurological disorders, Diseases

## Abstract

Myotonic dystrophy, or dystrophia myotonica type 1 (DM1), is a multi-systemic disorder and is the most common adult form of muscular dystrophy. It affects not only muscles but also many organs, including the brain. Cerebral impairments include cognitive deficits, daytime sleepiness, and loss of visuospatial and memory functions. The expression of mutated transcripts with CUG repeats results in a gain of toxic mRNA function. The antisense oligonucleotide (ASO) strategy to treat DM1 brain deficits is limited by the fact that ASOs do not cross the blood–brain barrier after systemic administration, indicating that other methods of delivery should be considered. ASO technology has emerged as a powerful tool for developing potential new therapies for a wide variety of human diseases, and its potential has been proven in a recent clinical trial. Targeting *DMPK* mRNA in neural cells derived from human induced pluripotent stem cells obtained from a DM1 patient with the IONIS 486178 ASO abolished CUG-expanded foci, enabled nuclear redistribution of MBNL1/2, and corrected aberrant splicing. Intracerebroventricular injection of the IONIS 486178 ASO in DMSXL mice decreased the levels of mutant *DMPK* mRNAs by up to 70% throughout different brain regions. It also reversed behavioral abnormalities following neonatal administration. The present study indicated that the IONIS 486178 ASO targets mutant *DMPK* mRNAs in the brain and strongly supports the feasibility of a therapy for DM1 patients based on the intrathecal injection of an ASO.

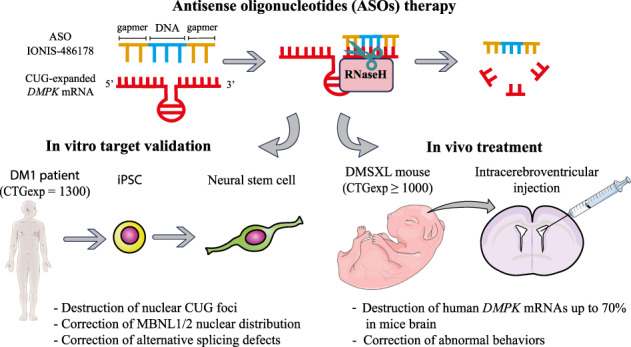

## Introduction

Myotonic dystrophy or dystrophia myotonica type 1 (DM1), also known as Steinert disease, was first described in 1909 by Steinert. It is a neuromuscular disorder that affects several organs and is the most common adult form of muscular dystrophy [1, 2]. DM1 is an autosomal dominant disease associated not only with progressive muscle weakness and myotonia but also with broad clinical features, including cardiac involvement, endocrine deficiencies, and central nervous system (CNS) alterations [1, 3, 4]. A peculiarity of this disease is a severe congenital form associated with ~20% neonatal mortality [5, 6], mental and intellectual disabilities, psychomotor disorders, and motor retardation in surviving infants [7–9]. Cerebral impairments observed in adult DM1 include anxiety, loss of visuospatial functions and memory, apathy, and daytime sleepiness. Overall, the quality of life of DM1 patients is drastically impacted by their cognitive deficits [10, 11]. Neurological afflictions are also associated with histopathological changes such as heterotopic neurons in the case of congenital DM1 [12, 13] and the aggregation of hyperphosphorylated fetal human TAU isoforms in adults, particularly in the hippocampus, amygdala, and entorhinal and temporal cortices [14, 15]. Young patients with congenital DM1 manifest severe brain structure abnormalities such as ventricular dilatation, cortical atrophy, and white matter lesions [12, 13, 16–18]. Neurodevelopmental defects are specific to early-onset DM1, while the CNS features of the adult form are reminiscent of neurodegenerative diseases [19].

DM1 is caused by an unstable expansion of CTG repeats in the 3’ UTR region of the dystrophia myotonica protein kinase (*DMPK*) gene located on chromosome 19q13.3 [20–23]. Unaffected individuals have 5–37 CTG repeats. When the repeat number exceeds 50 CTGs, this usually leads to disease manifestation, while larger repeat sizes are associated with an increase in the severity of symptoms. RNA transcripts containing CUG-expanded repeats (CUGexp-RNA) accumulate in nuclear aggregates (foci) that interfere with two families of RNA-binding proteins: CUGBP-Elav-like (CELF) and muscleblind-like (MBNL) proteins [24–28]. MBNL loss of function through sequestration in intranuclear RNA foci and CELF1 upregulation primarily perturb developmentally regulated splicing events. This not only results in the aberrant production of fetal isoforms in affected adult tissues [21, 29] but also affects RNA transcription, polyadenylation, and localization; protein translation; and miRNA processing [30–35]. The extent of these events in the CNS, how they contribute to neuropathogenesis, and their cellular specificity are not known [36].

Mouse [37–44], Drosophila [45–47], zebrafish [48, 49], and patient-derived induced pluripotent stem cell (iPSC) models are used to study the biological mechanisms underlying the DM1 pathology and to test possible therapeutic strategies [50]. Recent studies have confirmed DM1 phenotypes in iPSCs [50–53] and have made it possible to study the pathology using several DM1 cell types, some of which are less accessible than others. Several transgenic mice models have also been generated to investigate the pathophysiology of DM1. The DMSXL mouse model was designed to express a mutant form of the human *DMPK* gene with more than 1000 CTG repeats [37, 38]. Homozygote DMSXL mice express toxic transcripts that disrupt muscle, heart, brain, and respiratory functions by forming RNA foci and generating mis-splicing events [32, 54–56]. The expression of mutated *DMPK* mRNAs in the CNS reduces short-term synaptic potentiation in DMSXL mice and affects behavior functions, resulting in low levels of exploratory activity, spatial memory impairment, increased anxiety, and anhedonia [32].

No curative treatment is available for DM1 patients. Advances in the understanding of DM1 pathophysiological mechanisms indicate that inhibiting intranuclear RNA foci is a valuable therapeutic strategy. It has been shown in preclinical studies that antisense oligonucleotides (ASOs) with gapmer designs induce the degradation of intranuclear RNA foci both in vitro and in vivo through the RNase-H mechanism [57–60]. ASOs are also being investigated as therapeutic agents for multiple diseases, including diabetes, hyperlipidemias, cardiovascular diseases, cancer, and neurodegenerative diseases. Some ASO chemistries such as MOE and cEt have been proven to be effective for heart and skeletal muscle delivery and for correcting muscle phenotypes and cardiac conduction defects after systemic administration in DM1 mouse models expressing expanded CUG-mRNAs [58, 59, 61]. These results led to the first DM1 clinical trial based on ASO strategies (clinical trial NCT02312011). A major limitation of ASOs is that they do not efficiently cross the blood–brain barrier following systemic administration [59], indicating that other delivery methods are necessary to correct CNS deficits in DM1. A proof of concept study based on the intrathecal delivery of ASOs has shown that ASOs are effective in treating spinal muscular atrophy (SMA) [62].

We evaluated the effectiveness of the IONIS 486178 ASO in correcting the DM1 phenotype in human neuronal cells derived from iPSCs as well as cognitive impairments in DMSXL mice. We also characterized the pharmacodynamics and toxicity of the IONIS 486178 ASO in the CNS.

## Materials and methods

### Derivation of iPS cell lines

Human fibroblast cells from a control individual as well as from a patient with infantile-onset DM1 and a severe intellectual disability were reprogrammed using the Cytotune™-iPS 2.0 Sendai virus reprogramming kit according to the manufacturer’s instructions (Thermo Fisher Scientific, Ottawa, ON, Canada). Briefly, 2 × 10^5^ fibroblasts were transduced in six-well plates with an MOI of 5 for the KOS and hc-Myc viruses and an MOI of 3 for the hKlf4 virus. Seven days after transduction, the fibroblasts were transferred onto irradiated mouse embryonic fibroblast cells (MEFs) in hES medium (DMEM/F12) containing 20% knockout serum replacement, 1% GlutaMax^TM^, 1% non-essential amino acids, and 0.1 mM 2-mercaptoethanol (all from Gibco, Grand Island, NY, USA) supplemented with 10 ng/mL of FGF2 (StemCell Technologies, Vancouver, BC, Canada). Colonies with an iPSC-like morphology were manually picked after ~20 days. iPSCs were expanded on Matrigel-coated plates (Becton Dickinson, Mississauga, ON, Canada) with mTeSR1™ (StemCell Technologies), passaged with 0.5 mM EDTA (Thermo Fisher Scientific) in D-PBS (Millipore Sigma, Oakville, ON, Canada) every 3–4 days, and cryopreserved in Cryostem™ Freezing Medium (StemCell Technologies). The cells were routinely checked for mycoplasma contamination using Venor^®^GeM Mycoplasma OneStep PCR detection kits (Minerva Biolabs, Berlin, Germany), and the identity of a reprogrammed sample was verified by STR analysis (Genome Quebec, QC, Canada).

For embryoid body (EB) formation, the iPSCs were dissociated into single cells using Accutase^®^ (Stemcell Technologies) and were transferred into standing 25-cm^2^ flasks. The cell cultures were suspended in mTeSR1 medium supplemented with 10 μM Y-27632 (LC Laboratories, Woburn, MA, USA). After 24 h, the medium was changed to EB medium (DMEM/F12, 20% knockout serum replacement, 1% non-essential amino acids, 1% GlutaMAX™, and 0.1 mM 2-mercaptoethanol) supplemented with 10 μM Y-27632. The EBs were cultured in suspension for 8 days, plated on 0.1% gelatin-coated six-well plates, cultured for another 8 days, and then analyzed for three-germ-layer differentiation.

### Neural differentiation

Neural differentiation was performed using the neural rosette method in AggreWell™ 800 microwell culture plates (Stemcell Technologies) according to the manufacturer’s instructions. Briefly, 3 × 10^6^ iPSC cells were added to the wells of the culture plates. The wells contained STEMdiff™ Neural Induction Medium (NIM, StemCell Technologies) supplemented with 10 μM Y-27632. The cells were centrifuged at 100 × *g* for 3 min and were then cultured for 5 days in the same media without Y-27632. EBs were then plated on Matrigel-coated plates and were cultured for 7 days. The resulting neural rosettes were collected using STEMdiff™ Neural Rosette Selection Reagent (StemCell Technologies) and were transferred to poly-L-ornithine- (15 µg/mL, Millipore Sigma) and laminin- (10 µg/mL, StemCell Technologies) coated six-well plates. Neural rosettes and neural progenitor cells (NPCs) were cultured for 7 days in NIM and were then treated with Accutase^®^ to produce single-cell suspensions. NPCs were cultured in STEMdiff™ Neural Progenitor Medium (StemCell Technologies) and were treated with Accutase^®^ to produce single-cell suspensions. They were differentiated by culturing them in STEMdiff™ Neuron Differentiation Medium (StemCell Technologies) for 7 days. Poly-ornithine-laminin-coated plates were seeded at 5 × 10^4^ cells/cm^2^, and the cells were cultured for 15 days in STEMdiff™ Neuron Maturation Medium (StemCell Technologies).

### Immunofluorescence

The cells were fixed in 4% paraformaldehyde for 15 min at room temperature, rinsed with phosphate-buffered saline (Millipore Sigma), permeabilized, and blocked with 2% BSA (Millipore Sigma) supplemented with 10% goat serum (Gibco) and 0.2% Triton X-100 (Millipore Sigma) for 30 min. They were incubated with primary antibodies for 1 h and then with secondary antibodies for 30 min (Alexa Fluor, Thermo Fisher Scientific). See Table S1 for a list of the antibodies used. Nuclei were stained with DAPI (0.1 μg/mL), and the cells were imaged using a Zeiss Axio Imager (Carl Zeiss Microscopy, Jena, Germany) microscope.

### Electrophysiological experiments

Action potentials (APs) of iPSC-derived neurons were recorded from spherical single cells that exhibited bright cell bodies and two or more neurites on day 15 post-differentiation in the current-clamp mode. The membrane voltage was clamped at −60 mV, and the APs were elicited using the following current-clamp steps: 0 pA for 200 ms followed by 5 pA-steps from 0 to 100 pA over 800 ms. The cell-attached patch-clamp in gap-free mode approach was used to record spontaneous APs. Patch electrodes were pulled from Corning 8161 borosilicate glass capillaries (Harvard Apparatus Canada, Saint-Laurent, QC, Canada) and were fire polished before the recordings. Patch electrodes were filled with an intracellular solution containing (in mM): 130 KMeSO_4_, 10 di-Na-phosphocreatine, 10 HEPES, 2 MgCl_2_, 2 Mg-ATP, and 0.4 GTP and have a resistance of 6–7 mΩ. The pH was adjusted to 7.3 with 1 N KOH. The bath solution contained (in mM): 154 NaCl, 5.6 KCl, 2 CaCl_2_, 1 MgCl_2_, 8 D-glucose, and 10 HEPES. The pH was adjusted to 7.3 with 1 N NaOH.

Na^+^ currents were recorded as previously described [63]. Briefly, we used low-resistance, fire-polished pipets (≈1 MΩ) filled with the following intracellular solution (in mM): 35 mM NaCl, 105 mM CsF, 10 mM EGTA, and 10 mM HEPES. The pH was adjusted to 7.3 with 2 M CsOH solution. The external solution contained (in mM): 150 NaCl, 2 KCl, 1.5 CaCl_2_, 1 MgCl_2_, 10 glucose, and 10 HEPES. The pH was adjusted to 7.4 with 2 M HCl solution.

APs and Na^+^ currents were recorded with an Axopatch 200 amplifier (Molecular Devices, San Jose, CA, USA) at room temperature using 4 cells per condition. The recordings were acquired using pClamp software (Molecular Devices). Electrical signals were filtered at 5 kHz, digitized at 10 kHz, and stored on a computer equipped with an analog-to-digital converter (Digidata 1300; Molecular Devices) for further analysis.

### Fluorescent in situ hybridization (FISH)

FISH was carried out as described previously [59]. Briefly, cells were grown on Matrigel-coated coverslips and were fixed with 4% formaldehyde for 15 min at room temperature. They were then permeabilized with 0.4% Triton X-100 (Millipore Sigma) and were rehydrated with a 50% formamide (Thermo Fisher Scientific)/2X SSC buffer (Millipore Sigma) solution for 5 min. The cells were then incubated for 2 h at 37 °C in hybridization buffer composed of 50% formamide, 2X SSC, 1 mg/mL of yeast tRNA (Millipore Sigma), 10% dextran sulfate (Thermo Fisher Scientific), 0.02% BSA, 2 mM vanadyl ribonucleoside complex (Millipore Sigma), and 1 ng/µL of 5′-Cy3-labeled (CAG)5 PNA probe (PNA Bio, CA, USA). Following two 30-min washes in 50% formamide/2X SSC solution at 37 °C, the samples were counterstained with 0.1 μg/mL of DAPI (Millipore Sigma) and were mounted with Fluoromount (Millipore Sigma). Images were acquired using an LSM 700 confocal microscope with a Zeiss Axio Imager (Carl Zeiss Microscopy). FISH quantification of 500 nuclei per condition was performed using ImageJ 1.47 (NIH) software.

### PCR

RNA was isolated from liquid nitrogen flash-frozen mouse brains for RT-qPCR. A crude extract was obtained by homogenizing and thawing tissues for 30 min at 55 °C in a proteinase K solution containing 10 mM Tris-Cl, pH 7.5 (Wisent Bioproducts, St-Bruno, QC, Canada), 10 mM EDTA (Millipore Sigma), 2% SDS (Roche Diagnostics, Laval, QC, Canada), 500 mM NaCl (Thermo Fisher Scientific), 1.5 mM MgCl_2_ (Thermo Fisher Scientific), and 500 mg/mL of proteinase K (Qiagen, Montreal, QC, Canada). A standard QIAzol (Qiagen) RNA tissue extraction was performed. The RNA yield was measured using a Nanodrop 2000c spectrophotometer (Thermo Fisher Scientific). RT-qPCR was performed using a SYBR Green I Master hot start reaction mix on a LightCycler 480 Instrument II (Roche Diagnostics). *Hprt1*, *Rpl13a*, *Tbp*, *OAZ1*, *RPS13*, and *SRP14* were used as *DMPK* (see Table S2 for primers) normalization controls. RNA integrity was confirmed on MOPS denaturing agarose gels. RNA was isolated from cells using QIAzol for RT-PCR. All RNAs were reverse transcribed using QuantiTect Reverse Transcription kits (Qiagen). Data were analyzed using the Pfaffl method. RT-PCR splicing of 3 replicates per condition was performed with HotStarTaq DNA Polymerase kits (Qiagen) using specific primers (Table S3) and the following conditions: 1 cycle at 94 °C for 1 min, 30 cycles at 94 °C for 30 s, 58 °C for 30 s, and one cycle at 72 °C for 1 min. The PCR products were resolved on Tris-borate-EDTA 1.5% agarose gels using RedSafe nucleic acid stain (Biotium, Burlington, ON, Canada). Bands were observed using an AlphaImager (Alpha Innotech) and were analyzed by densitometry using the Analyze-Gels function in ImageJ 1.47.

### Southern blot analysis

Genomic DNA was extracted from 2 × 10^6^ cells using FlexiGene DNA kits (Qiagen). The DNA (20 µg) was double digested with EcoRV and HindIII (New England Biolabs, Whitby, ON, Canada), isopropanol precipitated, separated by electrophoresis on a 1% agarose TAE gel, and transferred onto an Immobilon-NY + nylon membrane (Millipore Sigma). The DNA was hybridized overnight at 42 °C with a human ^32^P-labeled *DMPK* oligonucleotide probe and was visualized using a Typhoon Trio+ imager (GE Healthcare Life Sciences, Mississauga, ON, Canada).

### Cell transfection

The synthesis and purification of the IONIS 486178 ASO were carried out by Ionis Pharmaceuticals as described previously [64]. See Table S4 for the chemically modified oligonucleotides. DM1 NPCs were transfected using Lipofectamine 3000 reagent (Thermo Fisher Scientific) when they reached 80% confluence. The ASO was used at final concentrations ranging from 31.25 to 500 nM. All analyses were performed after 24 h on 5 replicates per dose.

### Animals

Experiments involving animals were approved by the local Animal Care and Use Committee of the Research Centre of the Centre Hospitalier Universitaire de Québec (CHUQ). DMSXL transgenic and wild-type (WT) mice were produced in our laboratory. Female and male heterozygous DMSXL mice with 1000–1600 CTG repeats were used at 4 months of age for most experiments, with the exception of 19-day-old mice for the behavioral analyses. The mice were randomized by equally distributing sexes and littermates between groups. Experimental group sizes were determined by a small-scale pilot experiment. No animals were excluded from the analysis, and the investigator was not blinded at any point during the experiments.

### Intracerebroventricular injection of the ASO

Intracerebroventricular (i.c.v.) bolus injections were performed as previously described [65]. The ASO was dissolved and diluted in sterile D-PBS and sterilized by filtration through a 0.22-µm filter. The DMSXL mice were anesthetized with isoflurane (4% in O_2_, 1 L/min) and were placed in a rat stereotaxic frame (Stoelting, Wood Dale, IL, USA) with ultra-precise manipulator arms (Stoelting). The isoflurane was increased to 2% in O_2_ at a flow rate of 0.5 L/min (ABBOTT, Mississauga, ON, Canada) through a P28 mouse gas anesthesia head holder. A craniotomy was performed with a 2-mm ball head drill bit to inject 5 µL of the ASO with a Neuros syringe (Hamilton, City, QC, Canada) equipped with a 33-G blunt point needle at a rate of 1 µL/s at the following coordinates: X = 1.1 mm, Y = −0.2 mm, and Z = −2.2 mm from the bregma. After 5 min, the needle was slowly withdrawn, and the skin incision was sutured.

The dose-response experimental group sizes were: Saline (*n* = 5), 9 µg of ASO (*n* = 5), 19 µg of ASO (*n* = 5), 38 µg of ASO (*n* = 4), and 75 µg of ASO (*n* = 5). The time-effect experimental group sizes were 5 mice per time point for the IONIS-486178 ASO and 5 mice for the saline per time point.

### Immunohistochemistry

The distribution of ASO in mouse brain tissues was assessed by immunochemistry as described previously [66]. Briefly, fixed brain sections from 3 mice per group were digested for 1 min at 37 °C with a solution containing 500 mg/mL of proteinase K (Qiagen), 10 mM Tris-Cl, pH 7.5 (Wisent Bioproducts), 10 mM EDTA (Millipore Sigma), 2% SDS (Roche Diagnostics), 500 mM NaCl (Thermo Fisher Scientific), and 1.5 mM MgCl_2_ (Thermo Fisher Scientific). They were then blocked with 3% BSA for 30 min. After two washes in phosphate-buffered saline, the samples were incubated with a polyclonal rabbit anti-ASO primary antibody (6651 Pan ASO; Ionis Pharmaceuticals Inc., Carlsbad, CA, USA) for 1 h at room temperature and then with a goat anti-rabbit IgG (HRP) secondary antibody. The DAB chromogen (Abcam, Toronto, ON, Canada) was applied for 5 min before nuclear counterstaining with hematoxylin.

### Blood chemistry

Blood from 5 mice per group was collected through the inferior vena cava and was transferred to serum separator tubes (Thermo Fisher Scientific). The blood was incubated at room temperature for 30 min to allow coagulation and was then centrifuged at 10,000 × *g* for 5 min. The serum was collected in new tubes and was stored at −20 °C for further analysis. Alkaline phosphatase (ALP), alanine transaminase (ALT), aspartate transaminase (AST), creatine kinase (CPK), and creatinine (CRE) levels were measured using Olympus reagents and an Olympus AU400e analyzer (Olympus, Richmond Hill, ON, Canada).

### Histological analysis

Liver and kidney tissues from 5 mice per group were harvested, fixed in 10% buffered formalin for 24 h, dehydrated in graded ethanol, embedded in paraffin, and stained using hematoxylin and eosin. The histopathological changes were evaluated by an animal pathologist from IDEXX BioAnalytics. The tissues were examined microscopically and changes were graded according to severity, using a standard grading system where 0 = no significant change, 1 = minimal, 2 = mild, 3 = moderate, and 4 = severe. Half-points were used to represent intermediate findings.

### Neonatal intracerebroventricular injections

On neonatal day 1 (P1), hypothermic anesthesia was induced by placing the neonatal mice on a cold aluminum plate in ice. Anesthesia was confirmed by a color change from pink to purple, squeezing of paws, and cessation of movement. The mice were then injected with 2 µL of the IONIS 486178 ASO (14 µg) or 2 µL of saline by i.c.v. bolus injections using a Hamilton Neuros syringe with a 33 G needle. The ventricular injection sites were located at the following coordinates: X = 0.8 mm, Y = 1.5 mm, and Z = −1.7 mm from the bregma. The mice were then placed on a warming pad and were allowed to regain movement before being returned to the dam cage.

### Open field test

Mouse activity was tested at 19 and 20 days in square open 40 × 40 cm wood boxes. The open field arena was divided into a grid of equal-sized areas for the visual scoring of activity in different zones. Locomotor activity was measured with an infrared photobeam detection system (Any Maze, IL, USA). Each mouse was subjected to two 30-min sessions at 24-h intervals. The following data were collected to assess horizontal animal activity: (a) distance traveled, time mobile, and average speed in the apparatus during each 30-min session and (b) the number of entries and duration of visits in the different zones of the open field arena. Group sizes were: WT-Saline (*n* = 13), DMSXL-Saline (*n* = 11), and DMSXL-IONIS 486178 (*n* = 11).

### Statistical analysis

All statistical analyses were carried out using PRISM8 software (GraphPad, CA, USA). Data distribution normality was confirmed using the D’Agostino-Pearson test for 8 or more values and the Shapiro–Wilk test for fewer than 8 values. When only two groups were compared, the equality of variance was measured using an *F* test. If the two data sets had identical variances, a standard two-tailed unpaired Student’s *t*-test was used. If not, the test was performed with Welch’s correction.

When 3 or more groups were compared, the equality of variance was measured using the Brown–Forsythe test. If the data sets had identical variances, an ordinary one-way ANOVA with Tukey’s multiple comparisons test was used. If not, a Brown–Forsythe ANOVA test with Games-Howell’s multiple comparisons test was performed.

An ordinary two-way ANOVA with Tukey’s multiple comparisons test was performed to determine the effect of two independent variables on a dependent variable.

All the statistical tests were performed using a 95% confidence interval, and the differences were considered significant below the 0.05% risk threshold (*P < 0.05, **P < 0.01, ***P < 0.001, ****P < 0.0001).

## Results

### DM1 NPC models for antisense oligonucleotide screening

**Fig. 1:**
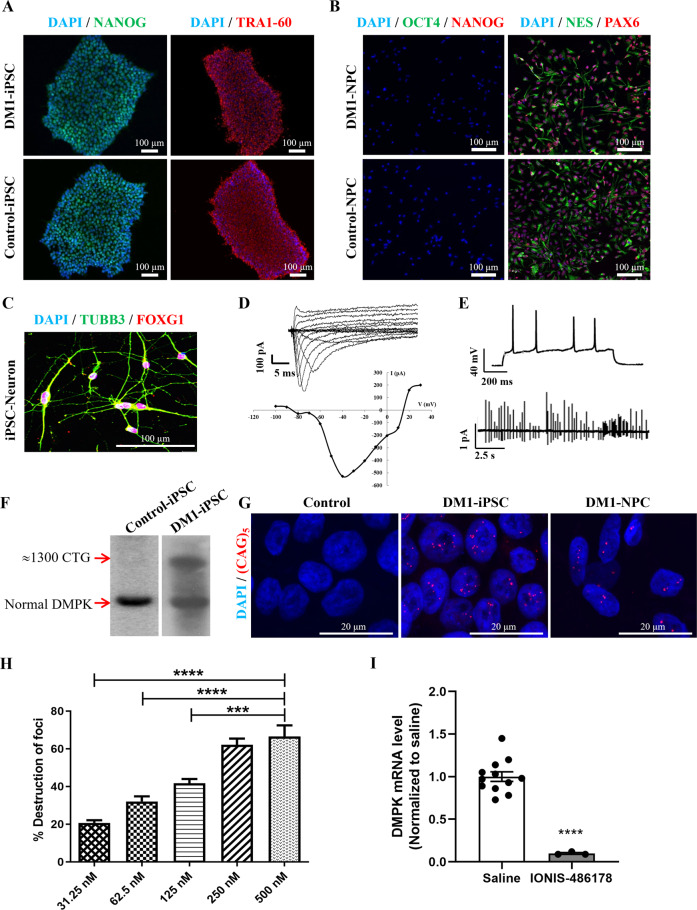
DM1 NPC model for antisense oligonucleotide screening. **A** Immunofluorescence of reprogrammed iPSC lines for the surface antigen TRA-1-60 and the nuclear pluripotency marker NANOG. **B** Loss of the pluripotency markers OCT4 and NANOG and expression of the neuroectodermal markers NES and PAX6. **C** Forebrain identity of neurons after 15 days of maturation. Patch-clamp measured **D** sodium currents, and **E** action potentials and cell-attached recordings of spontaneous cell firing activity. **F** Southern blot of DM1 iPSCs with a large repeat expansion in the *DMPK* gene. **G** FISH showing the presence of CUG-expanded foci in DM1 iPSCs and NPCs. **H** Quantification of overall CUG foci by FISH. Statistical analyses were performed using an ordinary one-way ANOVA with Tukey’s multiple comparisons test. **I** RT-qPCR *DMPK* mRNA analysis after a treatment with 500 nM IONIS 486178 ASO. A two-tailed unpaired Student’s *t*-test with Welch’s correction was used to determine the significance between the two groups. The error bars are presented as the mean ± SEM. SEM standard error of the mean.

Human fibroblast cells from a patient with infantile-onset DM1 and a severe intellectual disability and from a control patient were transformed into iPSCs using the Sendai virus technique employing Yamanaka factors (OCT4, KLF4, MYC, and SOX2). The established DM1 (1300 repeats) and control iPSC lines exhibited a typical human stem cell-like morphology under phase contrast microscopy (Fig. S1A). iPSC pluripotency was confirmed by immunostaining for the expression of the OCT4 and NANOG transcription factors, as well as the TRA-1-60, TRA-1-81, and SSEA-4 surface markers (Figs. 1A and S1A). The expression of the *OCT4*, *NANOG*, *DNMT3B*, *TERT*, and *REXO1* genes, as determined by RT-PCR, was similar in the human DM1 and control iPSC lines (Fig. S1B). A G-banding analysis showed that there were no karyotypic abnormalities in the human DM1 and control iPSCs (Fig. S1C). To confirm the pluripotency of the human iPSCs, we examined their capacity to spontaneously differentiate into the three germ layers. After 16 days in culture, attached cells showed various types of morphology, including those resembling neuronal cells and epithelial cells. RT-PCR confirmed that these differentiated cells expressed lineage-specific markers: *TUBB3* and *PAX6* (ectodermal lineage), *PECAM1*, *KDR*, and *GATA2* (mesodermal lineage), and *GATA4* and *AFP* (endodermal lineage) (Fig. S1D). These results showed that these iPSCs can differentiate into the three germ layers in vitro with no notable differences between the human DM1 and control iPSC lines.

Forebrain neurons were generated from DM1 and control iPSCs to establish in vitro DM1 models, as previously described by Xia Guangbin [51]. Immunostaining performed at different stages of iPSC differentiation showed that the endogenous pluripotency genes OCT4 and NANOG were downregulated in NPCs (Fig. 1B) while the genes involved in neuronal development (NESTIN and PAX6) were upregulated. In addition, more than 80% of the cells were positive for TUBB3 after neuronal maturation. Clusters of differentiated neurons expressed FOXG1, suggesting that these neurons had a forebrain identity (Fig. 1C). One of the clear changes observed as the neuronal cultures matured was their ability to repetitively fire APs during an 800-ms depolarizing current step. After 15 days of maturation, the neurons had acquired the ability to generate sodium currents and AP trains and exhibited spontaneous synaptic activities (Fig. 1D, E), confirming their normal biophysical properties.

To determine whether our DM1 in vitro model retained the characteristics of the disease, we assessed its DM1 molecular phenotype. A Southern blot analysis was carried out using genomic DNA isolated from cultured DM1 iPSCs. The blot showed the presence of a large expansion of ~1300 CTG repeats in the *DMPK* gene (Fig. 1F). Furthermore, FISH confirmed the presence of CUG-expanded foci in DM1 iPSCs and NPCs (Figs. 1G and S2). As reported in the literature [67], we also observed a certain variability in the number of nuclear foci, with some DM1 cells containing cytoplasmic CUG foci. This was attributed to different cell cycle stages. During mitosis and with the disruption of the nuclear envelope, most RNA foci disappeared. After cytoplasmic division of the cells, the foci were distributed randomly in their daughter cells. When the nuclear membrane reformed, the foci were either enveloped by the nuclear membrane or remained in the cytoplasm.

We previously identified a 16-nucleotide ASO gapmer (IONIS 486178) that contained cEt modifications targeting exonic regions of *DMPK* mRNA [59]. Our in vitro strategy consisted of performing a multiple dose evaluation of this ASO to identify the optimal concentration for reducing *DMPK* mRNA levels. Human DM1 NPCs were transfected with 31.25, 62.5, 125, 250, or 500 nM of the IONIS 486178 ASO. A quantitative analysis of overall CUG foci performed 24 h after the treatment revealed a dose-dependent reduction of foci in human DM1 NPCs (Fig. 1H), with a maximum 70% reduction at 500 nM. This reduction was associated with the reduction of *DMPK* mRNAs induced by the IONIS 486178 ASO (Fig. 1I).

### Reversal of the DM1 molecular phenotype in DM1 NPCs

**Fig. 2:**
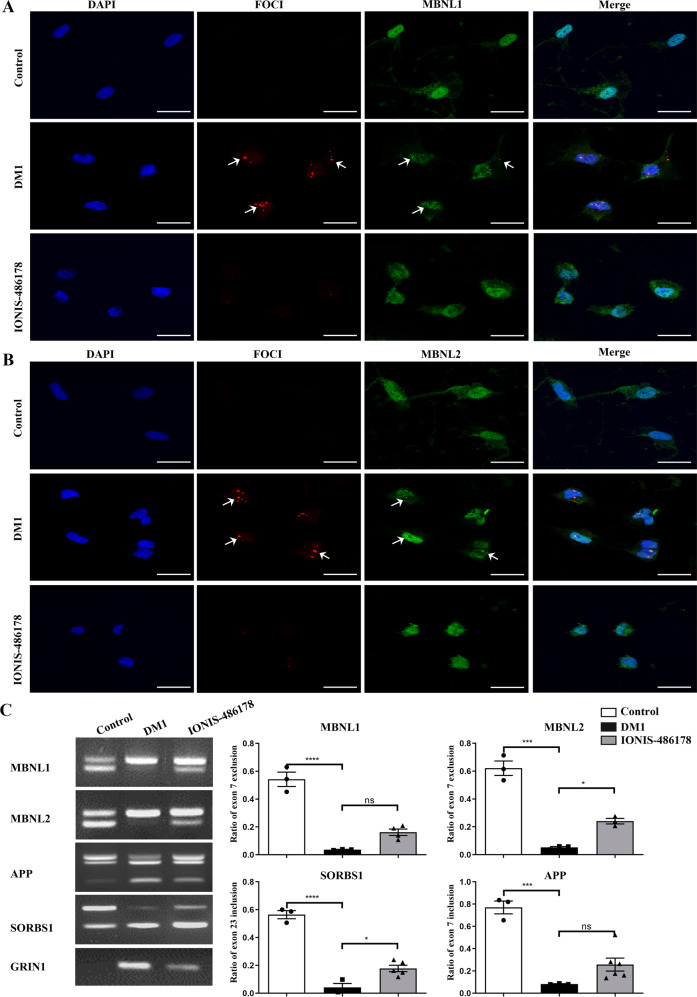
Correction of NPC DM1 phenotypes after a treatment with the IONIS 486178 ASO. **A** FISH immunofluorescence showing the destruction of nuclear foci and the redistribution of MBNL1 and **B** MBNL2 24 h after transfection with 500 nM IONIS 486178 ASO. **C** RT-PCR of DM1 alternative mis-splicing. Statistical analyses were performed using an ordinary one-way ANOVA with Tukey’s multiple comparisons test. The error bars are presented as the mean ± SEM. Scale bar: 20 µm.

We performed MBNL1 and MBNL2 immunostaining followed by a FISH targeting the CUG repeats in NPCs. We observed a diffuse distribution of MBNL1 and MBNL2 throughout the nuclei of control cells, whereas DM1 cells displayed a dotted pattern that colocalized with RNA foci, supporting the hypothesis that these splicing regulators are sequestered in DM1 NPCs (Fig. 2A, B). One day after a 500-nM IONIS 486178 ASO treatment, we observed that the number of RNA foci in DM1 NPCs was significantly reduced (Fig. 2A, B). A nuclear redistribution of MBNL1 and MBNL2 also occurred, suggesting that CUGexp repeats were successfully destroyed after cleavage events. We subsequently determined whether this reduction was sufficient to rescue the mis-splicing of *MBNL1*, *MBNL2*, *APP*, and *GRIN1*, which have been reported as DM1 neurological alterations and to be some of the genes with the highest splicing disruptions in human DM1 neuronal cells [68, 69]. We observed a partial rescue of the *MBNL1* and *MBNL2* exon 7 exclusion and of the *APP* exon 7, *GRIN1* exon 4, and *SORBS1* exon 23 inclusions, which is consistent with the reduction of RNA foci and redistribution of MBNL1 and MBNL2 (Fig. 2C).

### Potency of the IONIS 486178 ASO in the DMSXL mouse brain

**Fig. 3:**
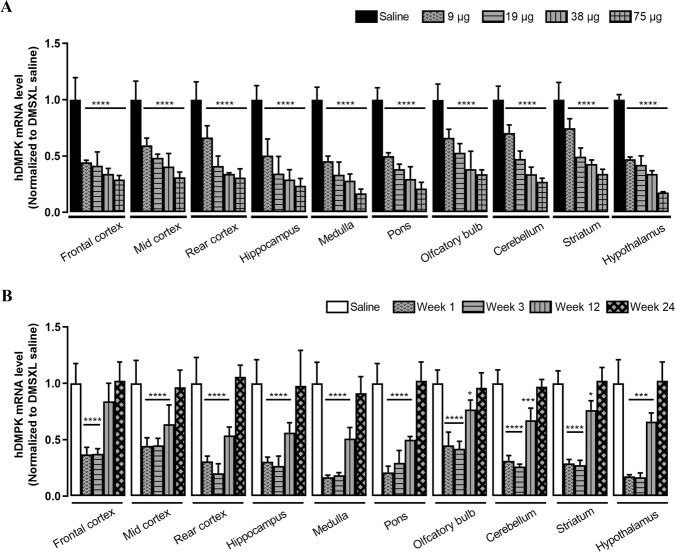
Intracerebroventricular injection of the IONIS 486178 ASO in DMSXL mice. RT-qPCR h*DMPK* mRNA analysis of a dose-dependent (**A**) and a time-dependent (**B**) ASO treatment in different brain areas. An ordinary two-way ANOVA with Tukey’s post hoc analysis for comparing multiple groups was used. The error bars are presented as the mean ± SD. SD standard deviation.

We evaluated the efficacy of the IONIS 486178 ASO for reducing human *DMPK* mRNAs in the brain following an i.c.v. injection in adult heterozygous DMSXL mice. The ASO was prepared in phosphate-buffered saline and was administered by i.c.v. using a single bolus injection in the right lateral ventricle of the brain. Five mice of mixed genders were treated with 9, 19, 38, or 75 µg of ASO. Their brains were dissected into 10 regions after 7 days for RNA analysis. An RT-qPCR analysis showed that there was a dose-dependent reduction in human *DMPK* mRNA levels throughout the brain (Fig. 3A). This reduction varied depending on the brain region, with a greater reduction at lower doses for the medulla, pons, hypothalamus, and hippocampus (more than 80% for 75 µg and 70% for 38 µg). We observed a decrease in human *DMPK* mRNA expression in the cortex, which did not exceed 70% with 75 µg of the IONIS 486178 ASO. These results demonstrated that the IONIS 486178 ASO is efficiently delivered by i.c.v. to different brain areas of DMSXL mice is efficient and that it can reduce *DMPK* mRNA expression in vivo.

To determine the duration of action of the IONIS 486178 ASO in the CNS, we followed its effect on human *DMPK* mRNA levels over 6 months. Heterozygous DMSXL mice of mixed genders received an i.c.v. bolus injection of 75 μg of the IONIS 486178 ASO. Five animals were sacrificed at various times after the injection. An RT-qPCR analysis showed that the maximum effect, which ranged from a 55% to an 83% reduction in human *DMPK* mRNAs, depending on the brain region, was maintained for at least 3 weeks after a single injection (Fig. 3B). mRNA knockdown was reduced by 30–50% 12 weeks after the injection and returned to untreated levels after 24 weeks, suggesting that this ASO is stable for 3–12 weeks in CNS tissues.

### Brain distribution of the IONIS 486178 ASO

**Fig. 4:**
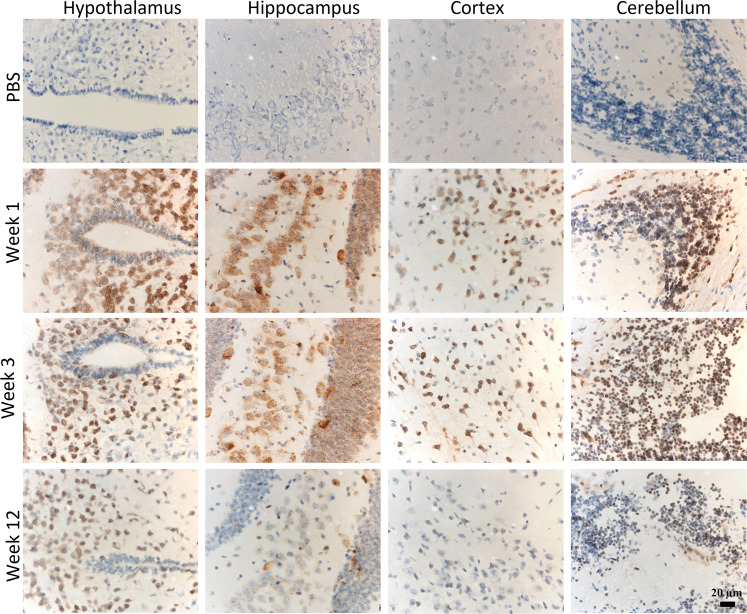
Mouse brain distribution of the IONIS 486178 ASO. Distribution of the ASO in the DMSXL mouse brain 1, 3, and 12 weeks after a 75 μg i.c.v. bolus injection. The ASO was visualized by immunostaining with an anti-ASO antibody (6651 Pan ASO) followed by counterstaining with hematoxylin. The scale bar of all images is shown on bottom right image.

We assessed the distribution of the IONIS 486178 ASO in the brain starting from the site of injection. Fixed brain sections were stained with a polyclonal antibody (6651 Pan ASO) that specifically recognizes phosphorothioate-modified ASOs [70]. The IONIS 486178 ASO exhibited broad brain distribution, with the greatest accumulations in the hypothalamus, hippocampus, cortex, and cerebellum. IONIS 486178 ASO diffusion to the white and gray matter was consistent (not shown). The IONIS 486178 ASO was detected in a time-dependent manner, with most of the ASO accumulating in the cytoplasm. The IONIS 486178 ASO was partially eliminated after 12 weeks of treatment (Fig. 4).

### The IONIS 486178 ASO does not cause CNS inflammation

**Fig. 5:**
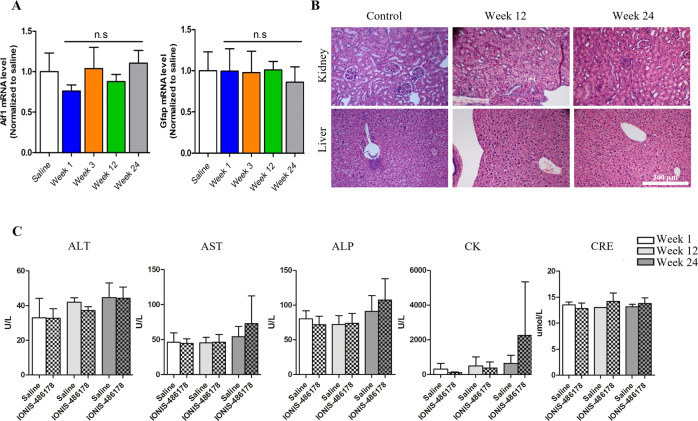
IONIS 486178 ASO toxicity profile in DMSXL mouse. **A** *Aif1* and *Gfap* mRNA expression profiles 1, 3, 12, and 24 weeks after a 75 μg i.c.v. bolus injection. Liver and kidney histology (**B**), the scale bar of all images is shown on bottom right image. Blood chemistry (**C**). ALP alkaline phosphatase, ALT alanine transaminase, AST aspartate transaminase, CK creatine kinase, CRE creatinine. Statistical analyses were performed using an ordinary one-way ANOVA with Tukey’s multiple comparisons test. The error bars are presented as the mean ± SD.

To determine the potential toxicity of the IONIS 486178 ASO in adult mice, we first determined whether the delivery by an i.c.v. bolus injection would result in the induction of astroglial (*Gfap*) and microglial (*Aif1*) activation, which plays an important role in the immune response [71]. The expression of *Aif1* and *Gfap* in CNS tissues was examined by RT-qPCR. We observed no increase in *Aif1* or *Gfap* mRNA levels in the brain after various treatment times, indicating that there was no significant increase in the number of astroglial and microglial cells (Fig. 5A).

We also investigated whether there was any peripheral toxicity when the IONIS 486178 ASO diffused from the CNS into the systemic circulation. A blood biochemistry analysis showed that the plasma markers ALT, ALP, and AST all remained within normal limits at 1-, 12- and 24-weeks post-injection. Similarly, CRE and CPK levels, which are markers for kidney and tissue damage, respectively, remained within normal thresholds during the experiment (Fig. 5C). Liver and kidney sections examined by an animal pathologist were all normal after 12 and 24 weeks of treatment (Fig. 5B). However, a mild renal pelvic inflammation as well as low numbers of mixed inflammatory cells infiltrating the gall bladder lamina propria were observed. Overall, the IONIS 486178 ASO seemed to be very well tolerated in adult mice following a single 75-µg i.c.v. injection.

### The IONIS 486178 ASO corrects behavioral abnormalities of DMSXL mice

**Fig. 6:**
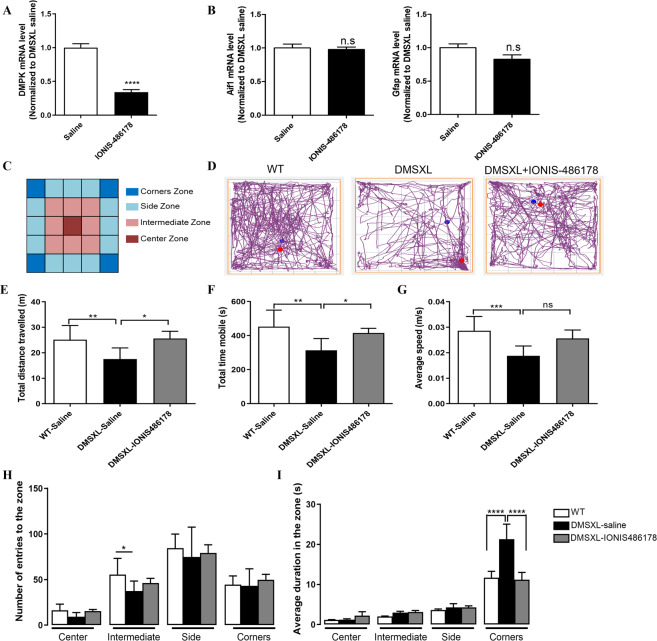
Behavioral abnormality correction of DMSXL mice after an IONIS 486178 ASO treatment. RT-qPCR h*DMPK* (**A**) and *Aif1* and *Gfap* (**B**) mRNA analysis of the brain. Schematic zones in the open field arena (**C**) and representative traces of mouse movement (**D**). Total distance traveled (**E**), time mobile (**F**), and average speed (**G**) of DMSXL homozygote mice. Number of entries into the zone (**H**) and duration of visit in the zone (**I**) of the open field arena. The statistical analyses were performed using a standard two-tailed unpaired Student’s *t*-test for **A** and **B**, an ordinary one-way ANOVA with Tukey’s multiple comparisons test for **F**. a Brown–Forsythe ANOVA test with Games-Howell’s multiple comparisons test for **E** and **G** and an ordinary two-way ANOVA with Tukey’s multiple comparisons test for **H** and **I**. The error bars are presented as the mean ± SEM for **A** and **B** or ± SD for **E**–**I**.

Homozygous but not heterozygous DMSXL mice (19–21 days old) have a behavioral phenotype of reduced exploratory activity and increased anxiety, which resembles a myotonic dystrophy type 1 neurological manifestation (Fig. 6D). To evaluate the efficiency of the IONIS 486178 ASO in correcting DM1 brain deficits, we first assessed the effects of a neonatal i.c.v. IONIS 486178 ASO injection in homozygous DMSXL mice. The ASO was prepared in phosphate-buffered saline and was administered as a single i.c.v. bolus injection. Thirteen mice of mixed genders were treated with 14 µg of the IONIS 486178 ASO at P1. The brains were harvested after 21 days for mRNA analysis and exhibited a 66% reduction in human *DMPK* mRNA levels (Fig. 6A), with no increase in the expression of *Aif1* or *Gfap* (Fig. 6B). Nineteen days after the treatment, the activity of the mice was assessed using the open field test. The number of entries and the duration of visits to the corner zones compared to the central zone of the open field arena indicated that there was a possible increase in anxiety of untreated DMSXL mice (Fig. 6H, I). They exhibited a significant decrease in exploratory activity, time mobility, and average speed shortly after their transfer into the open field arena (Fig. 6E–G), whereas the activity of mice treated with the IONIS 486178 ASO resembled that of WT mice, indicating that this ASO has the potential to correct behavioral abnormalities. To confirm that these results were not due to a correction of motor impairment, we examined the effect of the IONIS 486178 ASO on human *DMPK* mRNA levels in the tibialis anterior muscle after an i.c.v. injection. The amount of ASO injected had no effect on *DMPK* mRNA levels in the tibialis anterior muscle (Fig. S3). Moreover, the total dose injected in the CNS was very low compared to the dose necessary to target muscles.

## Discussion

In the present study, we evaluated the efficiency of the IONIS 486178 ASO for correcting the DM1 phenotype in human neural cells derived from iPSCs with 1300 CTG repeats. We showed that the IONIS 486178 ASO reduces nuclear foci, enables nuclear redistribution of MBNL1/2, and corrects aberrant splicing. Other studies using gene editing to reverse the mutation have reported similar phenotype reversals in iPSC-derived neurons [69, 72]. It was shown that early passage NPCs (less than 5 passages) may express both the fetal and adult *MBNL* isoforms and that the adult-to-fetal splicing ratio increases over time in culture [72]. In the present study, we used passage 4 NPCs. The physiological expression of fetal isoforms may thus explain why a minor but significant reversal of *MBNL* mis-splicing was observed in DM1 NPCs following a near complete suppression of total *DMPK* mRNAs by the ASO treatment. These results support the usefulness of iPSC-derived neural cells as a platform for screening potential drugs targeting CNS defects in DM1 brains.

We showed that the IONIS 486178 ASO was effective in targeting *DMPK* mRNAs, was well tolerated following an i.c.v. bolus injection, and resulted in an impressive downregulation of human *DMPK* expression throughout the DMSXL mouse brain. We observed the same long-lasting effects in the brain as previously reported for skeletal muscles [60], indicating that this ASO may be used at a similar dose-spacing interval. These data are in agreement with a previous study showing that ASOs exhibit prolonged activity in the CNS [65]. In addition, the brain, blood, liver, and kidney analyses suggested that the IONIS 486178 ASO does not exhibit any local or peripheral toxicity after a single i.c.v. injection. The effect of the IONIS 486178 ASO was previously evaluated in WT mice by subcutaneous injection, which demonstrated that the IONIS 486178 ASO also targets mouse *Dmpk* transcripts. The reduction in endogenous *Dmpk* mRNAs in WT mice caused no significant changes in mis-spliced RNAs and had no apparent deleterious effects on skeletal muscle or cardiac function [59, 73]. It is thus likely that the IONIS 486178 ASO also targets mouse *Dmpk* transcripts in the brain following an i.c.v. injection in DMSXL mice. It is, however, not known whether decreased levels of endogenous *Dmpk* mRNAs lead to a behavioral phenotype.

CUGexp-RNA nuclear aggregates have been identified in cortical and subcortical neurons in the brain [68]. As in skeletal [74] and cardiac muscles [75], these foci cause a dysregulation of alternative splicing regulators in the brain, inducing the production of aberrant isoforms of developmentally regulated genes in neurons and contributing to cognitive impairment in DM1 patients. In particular, the aberrant splicing of *MBNL1*, *MBNL2*, *APP*, *GRIN1*, and *MAPT* participate in the synaptic dysfunction and neurofibrillary degeneration seen in DM1 [15, 68]. Although they are not very easy to detect, the same splicing abnormalities have also been detected in DMSXL mice [32, 33]. Mbnl2 knockout mice develop significant brain defects, including cognitive deficits, impaired synaptic transmission, and spliceopathy, which testify to the contributing role of MBNL sequestration in DM1 CNS dysfunction [31, 76–79]. In the present study, the biodistribution of the IONIS 486178 ASO in the CNS after an i.c.v. injection was detected in the same areas as the foci [32], which correlates with a reduction in *DMPK* mRNA levels. This suggests that there is a nuclear redistribution of MBNL1 and MBNL2 and a correction of aberrant splicing, as observed in neuronal cells derived from iPSCs. These findings support the feasibility of a therapy based on the intrathecal administration of an ASO in DM1 patients like that of FDA-approved nusinersen administration for treating SMA.

The potential of the IONIS 486178 ASO for developing therapeutic strategies for congenital DM1 was evaluated after a neonatal i.c.v. injection. We hypothesized that a pre-emptive treatment with the IONIS 486178 ASO would correct behavioral and cognitive impairments. Our results showed that when the IONIS 486178 ASO is delivered to the developing brain, toxic mRNAs are reduced while behavioral abnormalities are significantly improved. As the ASO would be administered by an invasive procedure in human neonates, it would be advantageous to use a molecule with a half-life long enough to allow prolonged activity. Our preclinical data showed that the IONIS 486178 ASO is stable in CNS tissues and meets this criterion. We predict that neonatal IONIS 486178 ASO therapy has the potential to contribute to a therapeutic strategy for the treatment of congenital DM1 disease. A previous study using a type III SMA mouse model reported that earlier therapeutic interventions by embryonic i.c.v. injections are probably more effective than neonatal injections for phenotype correction [80]. However, in both strategies, ASOs led to 90% *SMN2* exon 7 inclusion in P7. Another study delivered an ASO targeting *MALAT1* RNAs by transuterine microinjection into mouse amniotic fluid at embryonic day 13 [81]. The ASO reduced target RNA expression for up to 4 weeks after birth and had a persistent effect on postnatal gene expression. To further improve therapeutic outcomes, the effects of the IONIS 486178 ASO will have to be evaluated after a single embryonic injection in utero compared to a neonatal i.c.v. injection.

The IONIS 486178 ASO significantly reduced mutant transcripts in neuronal cells in vitro and generated a robust reduction in h*DMPK* mRNA levels in different brain areas of DMSXL mice following an i.c.v. bolus injection. Our data support the development of an ASO therapy for DM1 brain deficits based on intrathecal injection. As this potential DM1 ASO therapy involves relatively invasive delivery methods such as intrathecal multi-dose injections, it is likely that it would have to be coupled with systemic injections to target skeletal muscles.

## Supplementary information

Supplementary information

## Data Availability

The data are available from the corresponding author on reasonable request.
