# Supplementary material for: Antisense oligonucleotides as a potential treatment for brain deficits observed in myotonic dystrophy type 1
Source: Gene Ther. 2022 Jan 25;29(12):698–709. doi: 10.1038/s41434-022-00316-7 (PMC9750879; doi:10.1038/s41434-022-00316-7)
Supplement: Supplementary file 1 — Supplementary information [file 41434_2022_316_MOESM1_ESM.docx]

**Supplementary information**

**
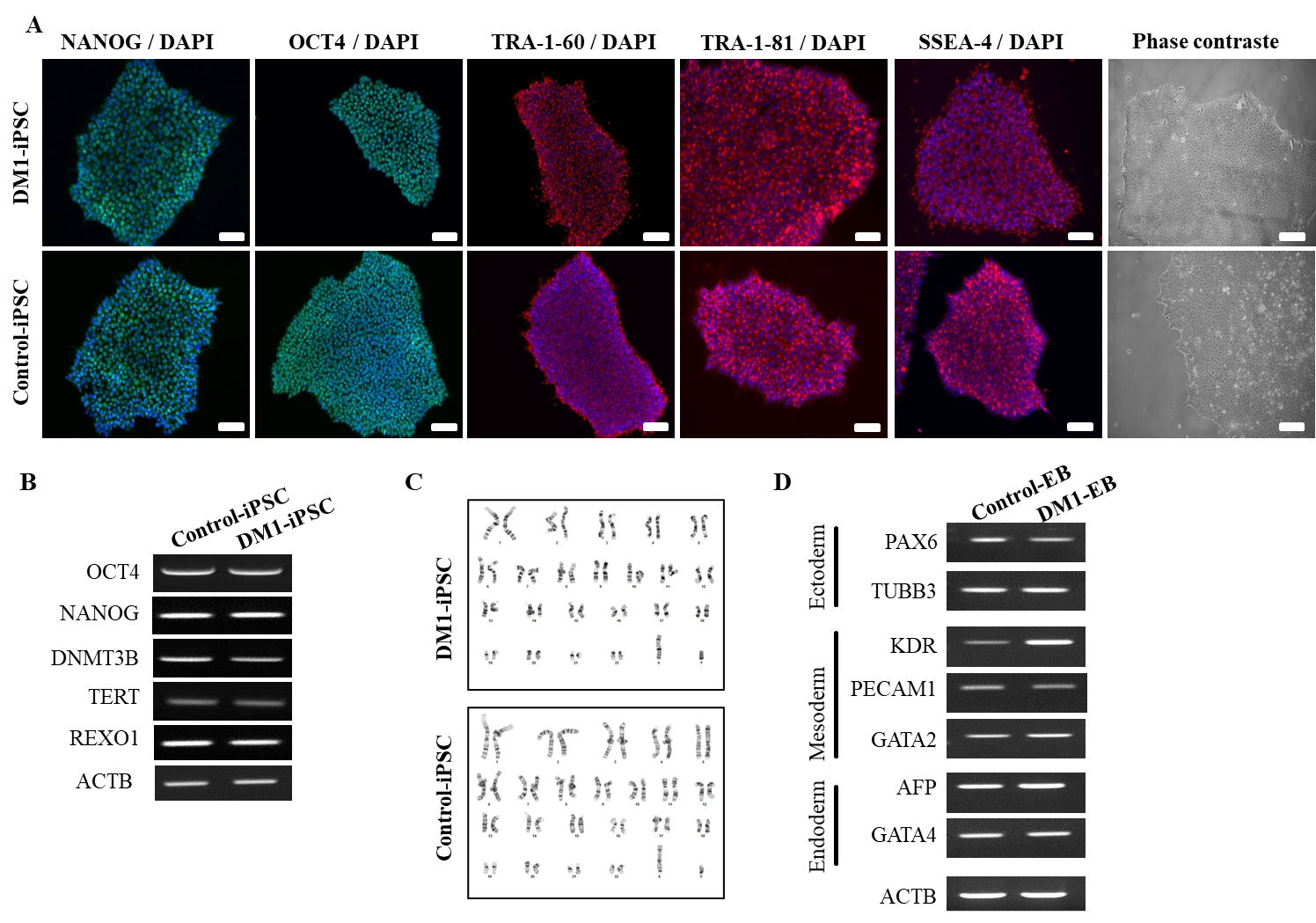
**

**Fig. S1. Characterization of DM1 iPSCs.** (**A**) Immunofluorescence of surface antigens TRA-1-60, TRA-1-81 and SSEA-4 and nuclear pluripotency markers OCT4 and NANOG. (**B**) RT-PCR of pluripotency markers with internal control ACTB. (**C**) G-banding karyotype analysis of iPSC lines. (**D**) RT-PCR for spontaneous differentiation capacity of embryonic bodies to endoderm, mesoderm and ectoderm germ layers. Scale bar :100 µm.

**
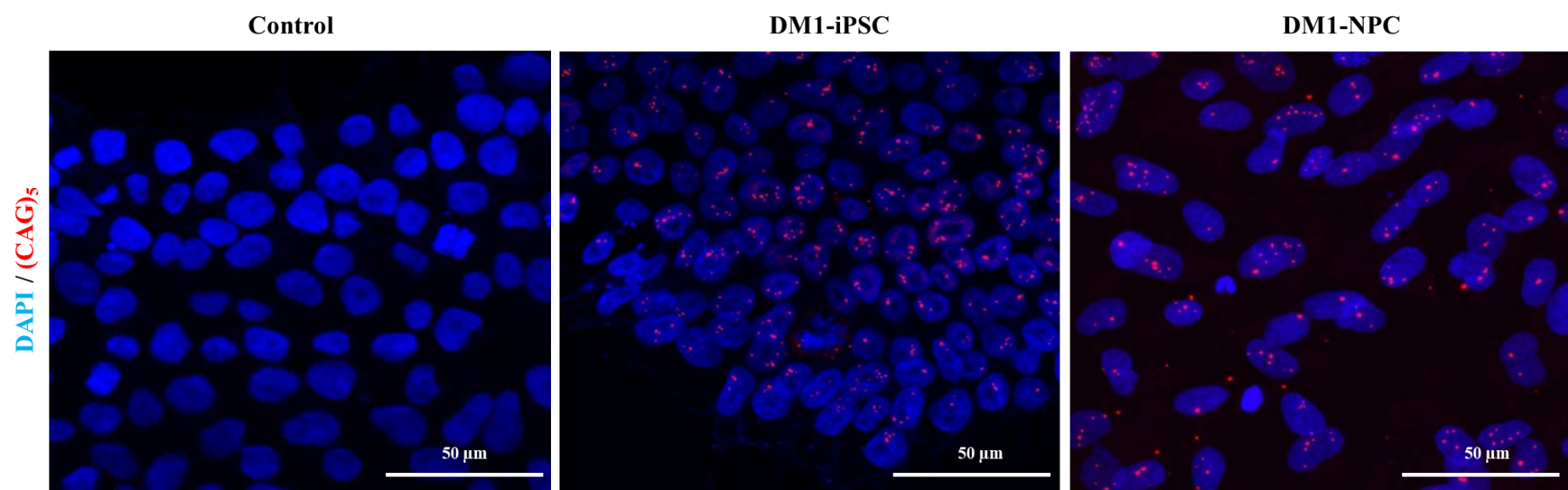
**

**Fig. S2. FISH images showing the presence of mainly nuclear foci in DM1 iPSCs and NPCs.**

**
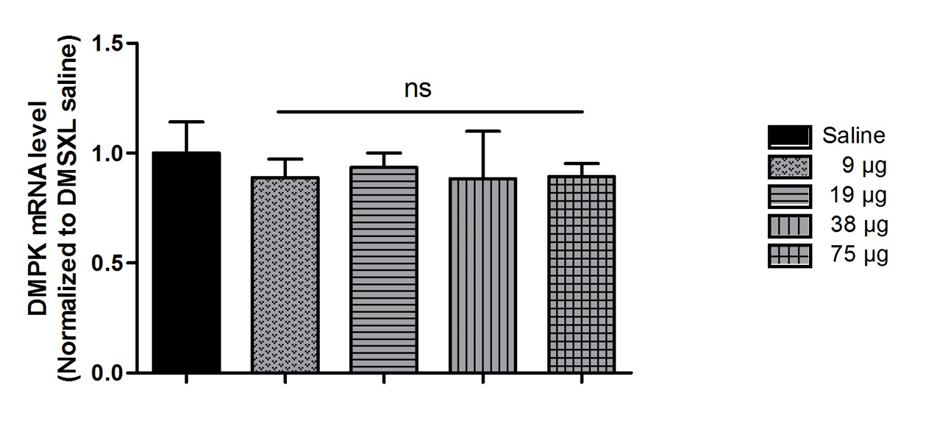
**

**Fig. S3. IONIS 486178 systemic bleed through muscle control from ICV injection.** DMSXL mice tibialis anterior muscle was tested for ASO bleed through from the ASO ICV injection. N = 5, ns: non-significant, One-way ANOVA statistical test with Tukey’s post hoc analysis for comparison between multiple groups. Data are presented as the mean ± SD

**Table S1**: Antibody details.

|  | Antibody | Company Cat # | Dilution |
| --- | --- | --- | --- |
| iPSC markers | OCT4 | Santa cruz, sc5279 | 1/500 |
|  | NANOG | Millipore, MABD24 | 1/2000 |
|  | SSEA-4 | Life technologies, 414000 | 1/100 |
|  | TRA-1-60 | Invitrogen, 411000 | 1/72 |
| NPC markers | PAX6 | Abcam, ab5790 | 1/200 |
|  | NES | Abcam, ab6320 | 1/500 |
| Neuron markers | TUBB3 | Abcam, ab14545 | 1/200 |
|  | FOXG1 | Abcam, ab18259 | 1/100 |

**Table S2**: RT-qPCR Primers details for hDMPK levels and reference genes.

|  | Target | Forward/Reverse sequence |
| --- | --- | --- |
| DMSXL mouse | hDMPK | 5'-CCTATCGTTGGTTCGCAAAGT-3' |
|  |  | 5'-CAAAAGCAAATTTCCCGAGTAA-3' |
|  | Hprt1 | 5'-GTTGGATACAGGCCAGACTTTGT-3' |
|  |  | 5'-CACAGGACTAGAACACCTGC-3' |
|  | Rpl13a | 5'-CCCTCCACCCTATGACAAGA-3' |
|  |  | 5'-CTGCCTGTTTCCGTAACCTC-3' |
|  | Tbp | 5'-GCCTTCCACCTTATGCTCAG-3' |
|  |  | 5'-GCTACTGCCTGCTGTTGTTG-3' |
|  | Aif1 | 5'-CTGGAGGGGATCAACAAGCAATTC-3' |
|  |  | 5'-CCAGCATTCGCTTCAAGGACATAA-3' |
|  | Gfap | 5'-GACCAGCTTACGGCCAACAG-3' |
|  |  | 5'-TTCATCTTGGAGCTTCTGCCT-3' |
| NPC cells | OAZ1 | 5'-AATAGCCACTGCTTCGCCAG-3' |
|  |  | 5'-GAGACCCTGGAACTCTCACT-3' |
|  | RPS13 | 5'-ATCTGACGACGTGAAGGAGC-3' |
|  |  | 5'-TCTCTCAGGATTACACCGATCT-3' |
|  | SRP14 | 5'-ACGTCGGGCAGCGTCTAT-3' |
|  |  | 5'-TCCTTGGAGCTCACCACAGT-3' |

**Table S3**: RT-PCR Primers details.

|  | Target | Forward/Reverse Sequence |
| --- | --- | --- |
| Pluripotency Markers | OCT4 | 5’-GTGGAGGAAGCTGACAACAA-3’ |
|  |  | 5’-CAGGTTTTCTTTCCCTAGCT-3’ |
|  | NANOG | 5’-TCCAACATCCTGAACCTCAG-3’ |
|  |  | 5’-GACTGGATGTTCTGGGTCTG-3’ |
|  | DNMT3B | 5’-ATAAGTCGAAGGTGCGTCGT-3’ |
|  |  | 5’-GGCAACATCTGAAAGCCATTT-3’ |
|  | TERT | 5’-TGTGCACCAACATCTACAAG-3’ |
|  |  | 5’-GCGTTCTTGGCTTTCAGGAT-3’ |
|  | REXO1 | 5’-TGGACACGTCTGTGCTCTTC-3’ |
|  |  | 5’-GTCTTGGCGTCTTCTCGAAC-3’ |
|  | PAX6 | 5’-TCTAATCGAAGGGCCAAATG-3’ |
|  |  | 5’-TGTGAGGGCTGTGTCTGTTC-3’ |
|  | TUBB3 | 5’-GATCGGAGCCAAGTTCTG-3’ |
|  |  | 5’-GTCCATCGTCCCAGGTTC-3’ |
|  | GATA4 | 5’-CTAGACCGTGGGTTTTGCAT-3’ |
|  |  | 5’-TGGGTTAAGTGCCCCTGTAG-3’ |
|  | AFP | 5’-AGCTTGGTGGTGGATGAAAC-3’ |
|  |  | 5’-CCCTCTTCAGCAAAGCAGAC-3’ |
|  | PECAM1 | 5’-CCCAGCCCAGGATTTCTTAT-3’ |
|  |  | 5’-ACCGCAGCATCATTTGAGTT-3’ |
|  | GATA2 | 5’-GCAACCCCTACTATGCCAACC-3’ |
|  |  | 5’-CAGTGGCGTCTTGGAGAAG-3’ |
|  | KDR | 5’-AGTGATCGGAAATGACACTGGA-3’ |
|  |  | 5’-GCACAAAGTGACACGTTGAGAT-3’ |
|  | ACTB | 5’-GGA CTT CGA GCA AGA GAT GG-3’ |
|  |  | 5’-AGCACTGTGTTGGCGTACAG-3’ |
| Splicing Markers | SORBS1 | 5'-CGGAAGAATTTATTCGAAGACG-3' |
|  |  | 5'-TGTAGGTGCGTGGGAAGATT-3' |
|  | MBNL1 | 5'-GCTGCCCAATACCAGGTCAAC-3' |
|  |  | 5'-TGGTGGGAGAAATGCTGTATGC-3' |
|  | MBNL2 | 5'-ACAAGTGACAACACCGTAACCG-3' |
|  |  | 5'-TTTGGTAAAGGATGAAGAGCACC-3' |
|  | APP | 5'-CCACCACCACAGAGTCTGTGGAA-3' |
|  |  | 5'-GACATTCTCTCTCGGTGCTTGGCCT-3' |
|  | GRIN1 | 5'-GTCTACAGCTGGAACCACATC-3' |
|  |  | 5'-TCCATCAGCAGGGCCGTCACG-3' |

**Table S4**: Antisense oligonucleotide sequences.

Phosphorothioate (**s**), β-D-2'-deoxyribonucleoside (**d**), 5-methylsytosine nucleoside (**^m^C**), 6'-(S)-CH3 bicyclic nucleoside (**k**), 2'-O-methoxyethyl nucleoside (**e**)

| Name | Chemistry | Sequence (5’-3’) |
| --- | --- | --- |
| IONIS-486178 | cEt | A_ks_^m^C_ks_A_ks_A_ds_T_ds_A_ds_A_ds_A_ds_T_ds_A_ds_^m^C_ds_^m^C_ds_G_ds_A_ks_G_ks_G_k_ |
